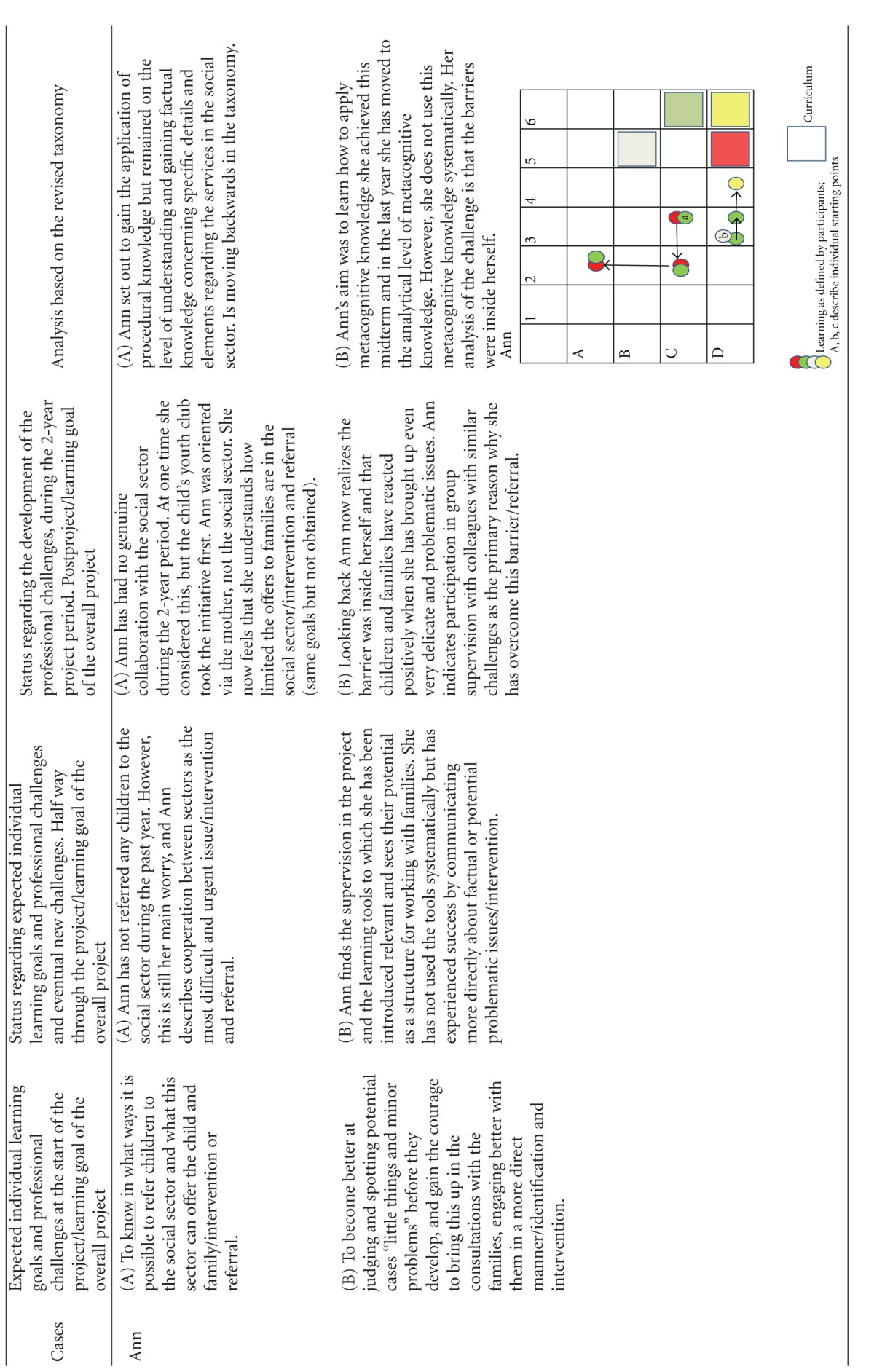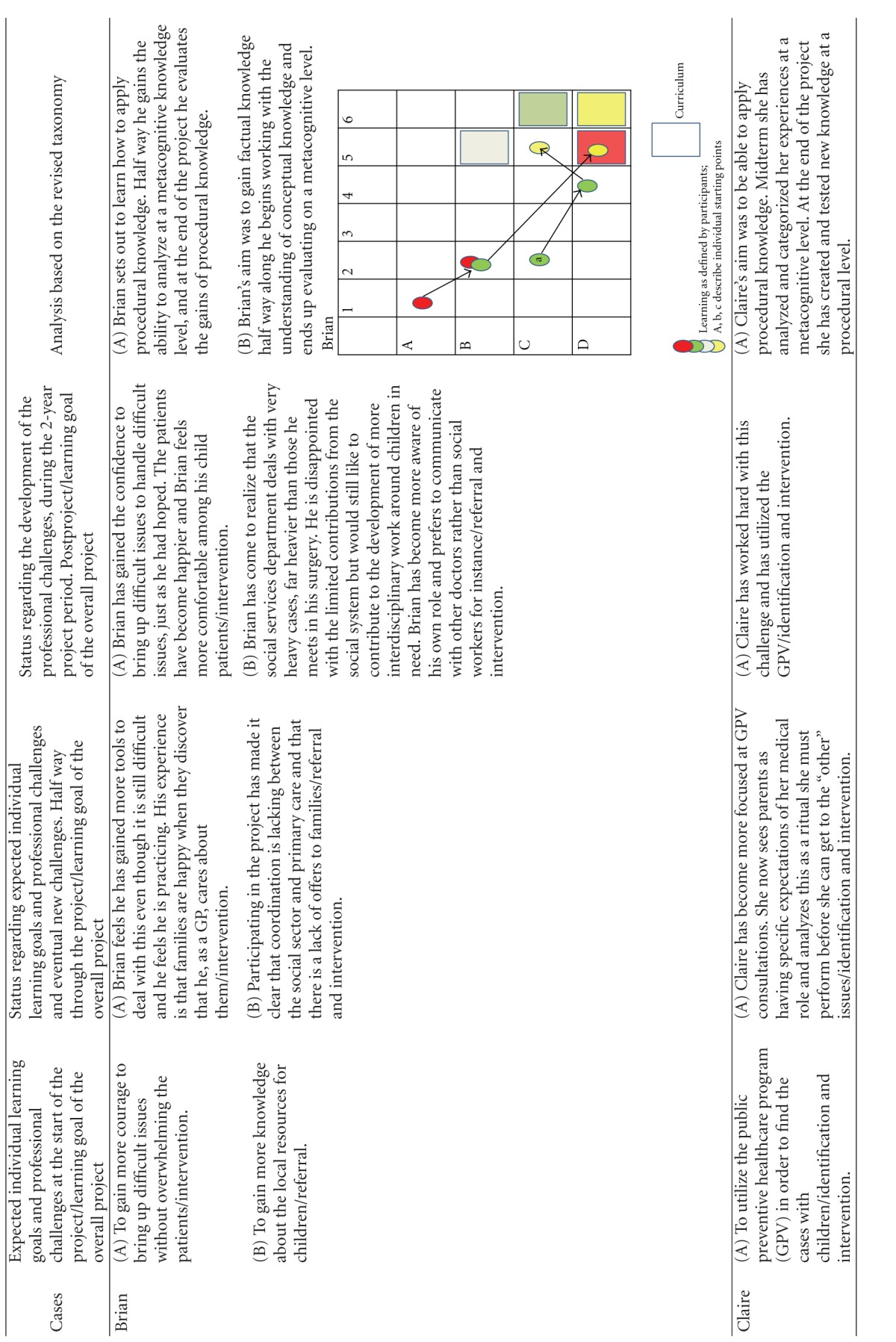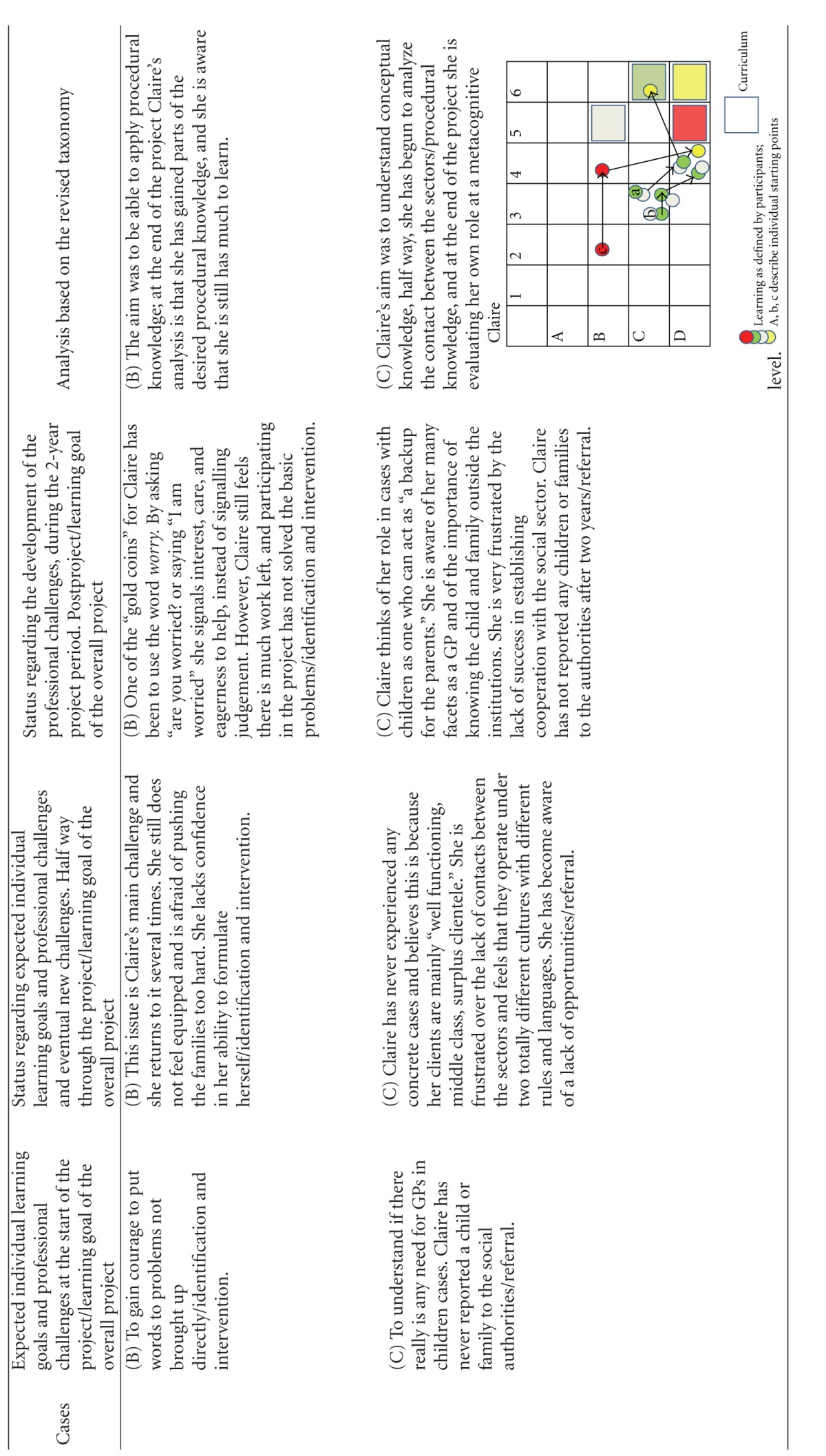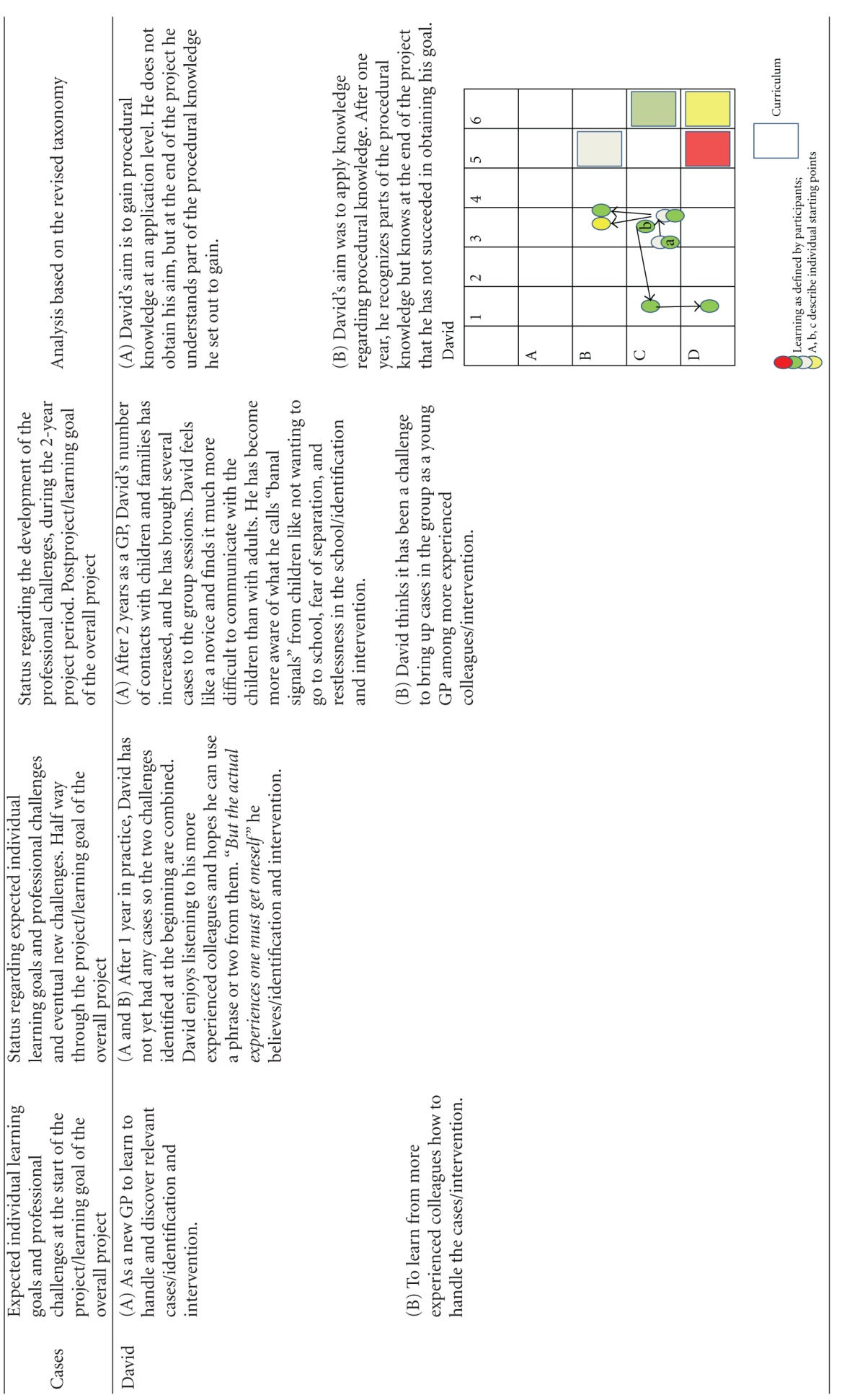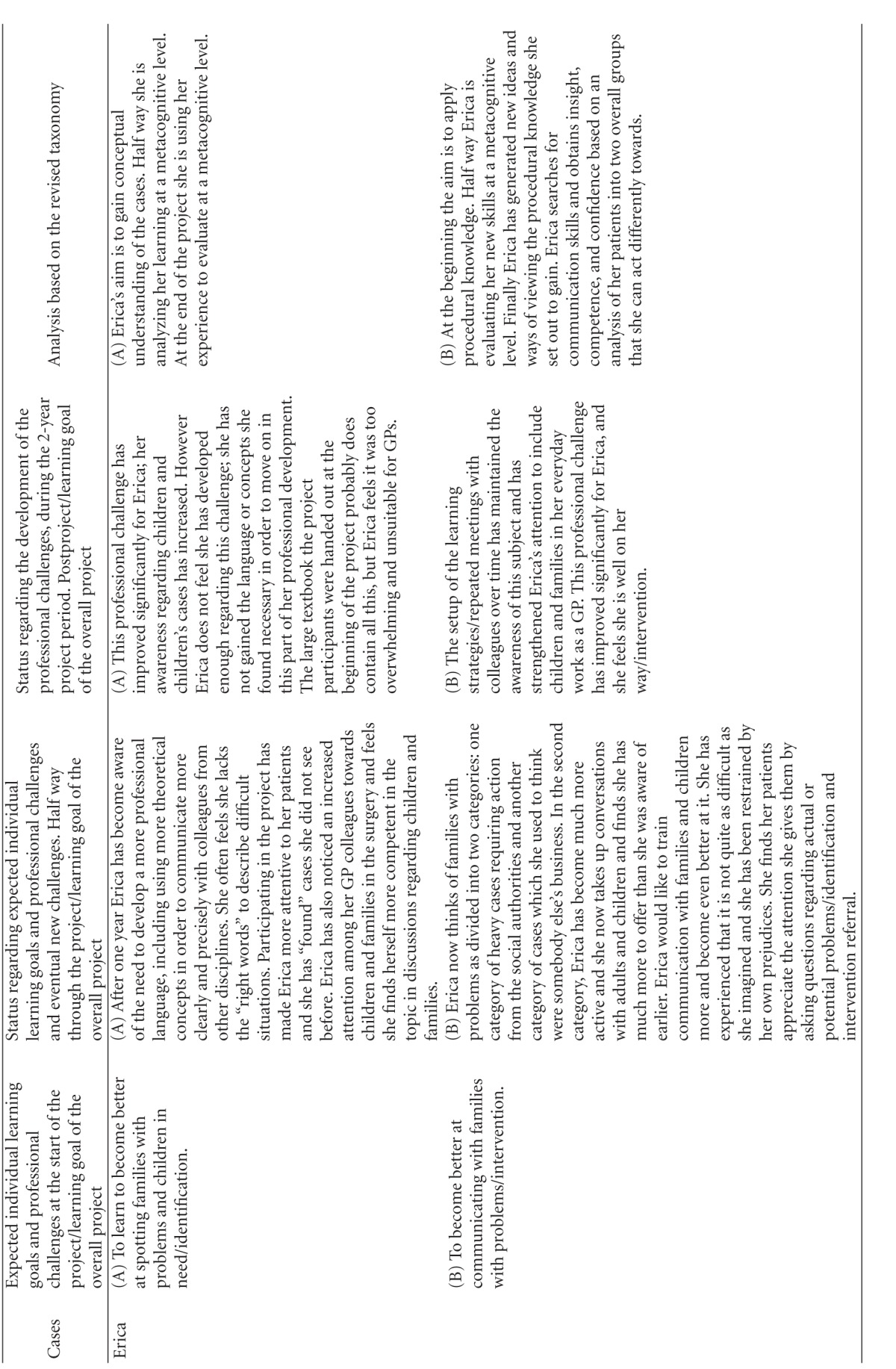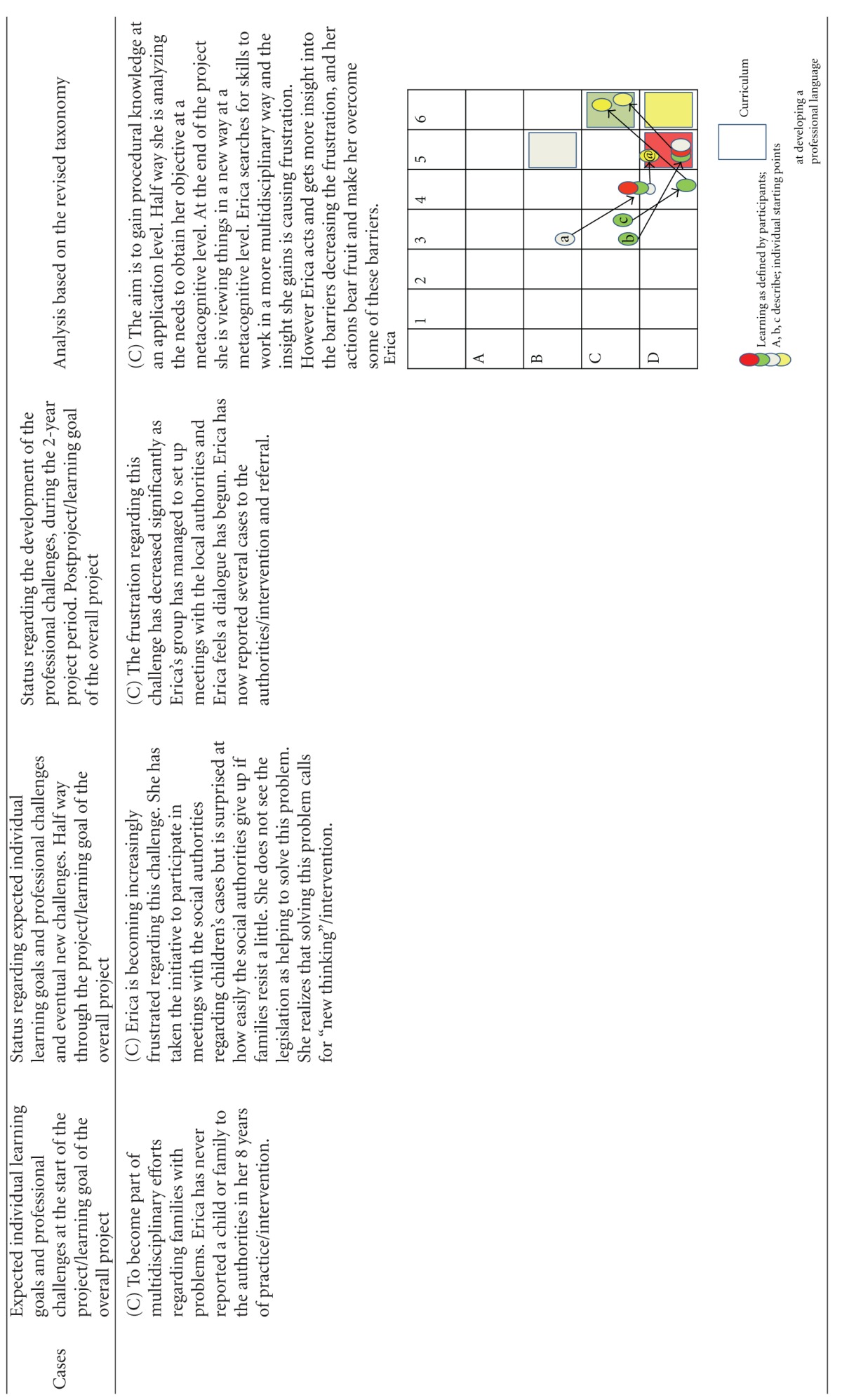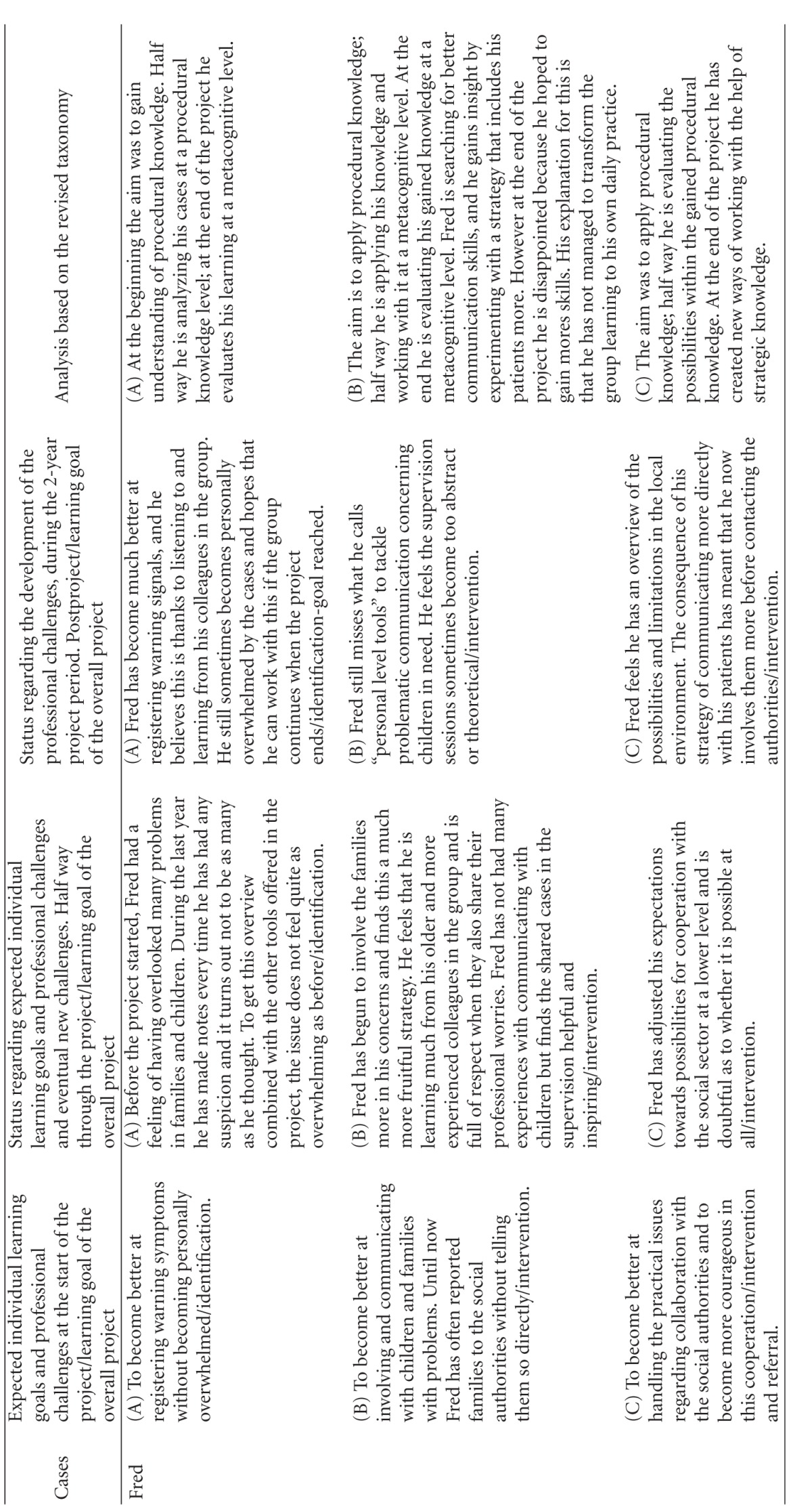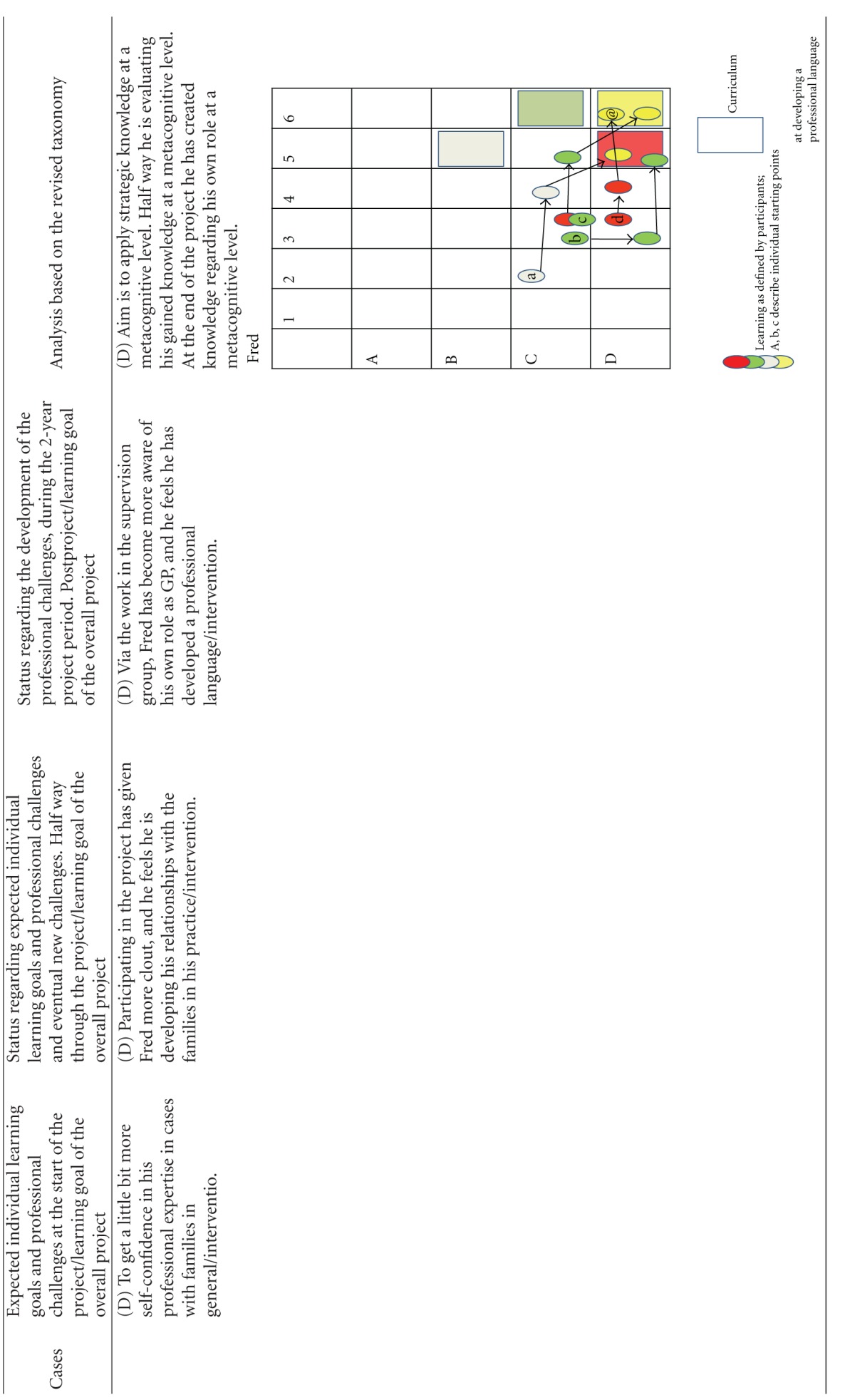# Individual Development of Professionalism in Educational Peer Group Supervision: A Multiple Case Study of GPs

**DOI:** 10.1155/2012/792018

**Published:** 2012-05-27

**Authors:** Bibi Hølge-Hazelton, Charlotte Tulinius

**Affiliations:** ^1^The Research Unit for General Practice and Section of General Practice, Department of Public Health, University of Copenhagen, 1014 Copenhagen, Denmark; ^2^Hospital North, Region Zealand, Denmark; ^3^St. Edmund's College, University of Cambridge, Cambridge CB3 0BN, UK

## Abstract

*Background*. Research has shown that peer-group supervision can strengthen GPs' professionalism, but little is known about the individual learning processes. To establish professionalism beyond professional behaviour, identity and idealism need to be included. The inner attitudinal values of professionalism within the individual are, however, difficult to assess. *Aim*. On the basis of a multiple case study, this paper describes the process of professional learning and challenges for individual GPs, as they take part in supervision groups focusing on children cases. *Methods and Results*. By using a two-dimensional theoretical model, it is shown that all GPs developed their professional behaviour, and many of them strengthened their professional identity in this domain towards a changed professionalism. Most participants emphasized the positive experience of sharing worries with families indicating care and interest. Some participants learning processes were very linear/convergent; others were complex/divergent—starting out with a relatively simple objective, realizing how multifaceted the issue was after the first year leading to a final development of new perspectives or action possibilities. *Conclusion*. The composition of supervision groups, as well as the professional background of the supervisor, may play a significant role in the development of professional behaviour and professionalism.

## 1. Introduction

Medicine is based on professional virtues such as self-regulation, autonomy authorisation, specialisation, and adherence to an ethical code of practice. The privilege of self-regulation assumes assurance of the competencies of every practicing doctor, which is gained by standards for education and practice [1]. 

The amount of expectations of what GPs are supposed to have knowledge about is large and ever developing, from the profession itself, from society, and from patients. Learning is expected to take place through continuing professional development (CPD), known to achieve the best outcome if integrated within daily clinical practice, performed over time, through a mix of activities and sources of knowledge, and, involving educational meetings [2–5].

It has been suggested that GPs need more knowledge regarding social, emotional, and cognitive development of young children [6]. They also need to be able to describe problems within the field to communicate effectively with other professionals in the development of a common language [6–8].

To obtain professional behaviour, knowledge needs to be applicable, and several models have been suggested (e.g., [7, 8]). To establish professionalism beyond professional behaviour, identity and idealism need to be included. The inner attitudinal values of professionalism within the individual are, however, difficult to assess [9].

Studies have shown that GPs perceive CPD as important [10]. There is, however, an ongoing debate on the assumption that doctors can identify and remedy any decencies in their own knowledge and skills, especially in relation to their status as reflective practitioners. This is particularly true for self-regulating professionals such as GPs, for whom CPD and the development of reflective practice are often left almost entirely to the individual.

Studies of supervision groups for GPs have been demonstrated to prove valuable in establishing a shared understanding, in conceptualizing children cases in general practice [11, 12]. These kinds of group studies contribute to the called-for development of a common language [6] and a broader understanding of the challenges in general practice while working with children and their families. However, it has not been possible to identify any published work regarding how the individual GP regards and responds to the professional challenges when confronted with clinical questions in supervision.

The aim of the paper is to describe the process of professional learning and challenges at the individual level among general practitioners, in this paper exemplified by a study where GPs took part in educational peer group supervision focusing on children cases.

## 2. Materials and Methods

### 2.1. Methods of Data Collection

A practice-based project was set up, in Denmark, from 2005 to 2007 by GPs with a special interest in child health, with the aim to prevent the neglect of children by early and competent action and to strengthen the professional identity of the participating GPs in children cases [11, 12]. The specific learning objectives were to strengthen the GPs' competencies in

- identification (of a child case),
- referral (of a child to relevant local initiatives or parts of the social and health care system),
- intervention (relevantly in a child case).

A case with a “child in need” in general practice is defined as “a case that directly or indirectly involves problems with a specific child, an as-yet unborn child, or one or both parents of a family, currently or potentially threatening the well-being of the family or the child” [11, 12].

The main intervention was the participation of 21 GPs in three peer groups, meeting regularly for educational supervision over a 2-year period, focusing on cases involving children from the GP's clinical practices. The supervision method was inspired by reflective team/peer groups [13].

Moreover, a number of other learning tools were offered to the participants: teaching days, written material, and electronic portfolios. The activities and the GPs; learning were followed in a multimethod evaluation by the authors [11].

The original intention was to find supervisors with a GP background, but for pragmatic reasons two groups were led by GPs with supervision training background and the third by a clinical psychologist/child expert.

### 2.2. Methods for the Analysis

The issue is complex and content dependant. Therefore, a multiple case study research design was set up focusing on the circumstances, dynamics and complexity among six of the twenty-one participants. The cases were explored in depth, retrospectively over a 2-year period through participant observations, interviews before, midterm, and after the project ended, and using a written evaluation questionnaire (described in [11]).

Selecting the case unit: one male and one female GP from the three different geographically groups: urban, suburban, and countryside, representing different practice organization forms: solo and shared, part-time and full-time, and number of years' of experience as GPs: 0–30 were selected before the interventions began.

Following the suggestions of Stake, in Bowling [14, page– 406], the analysis was done in the following four stages:

1. A chronological or biographical description of the cases.
2. The investigators' approach to understanding and investigating the cases.
3. A description of each, in turn, of the major components of the cases.
4. Finally, vignettes which describe particular episodes.

## 3. Results

### 3.1. Stage 1: A Chronological or Biographical Description of the Cases

Six cases are presented in Table 1 including information regarding gender, age, geography, number of years in practice, practice organization form, previous experience with participation in educational supervision groups as continuous professional development (CPD), and the professional background of the supervisors. Each participant was given a new name, and any person-identifiable parameters or information was deleted or changed.

### 3.2. Stage 2: The Investigators' Approach to Understande and Investigate the Cases

The focus is individual learning. Each participant described his/her learning objective before the intervention began, self-reported learning halfway and at the end of the project. To analyze the individual learning and the learning objectives of the overall project, we used the two-dimensional theoretical “model the revised taxonomies” [15] presented in Table 2.

Using this model, we first categorized the learning objectives. The overall learning objective for the project was categorized as “creation at a metacognitive level” (color coded yellow, and represented as a yellow square in the diagrams of Table 3). The learning object of “identification” was defined by the project designers to be obtained to the level of “evaluation of conceptual knowledge” (represented as a grey square in the diagrams of Table 3), the learning objective of “referring children” to the level of “evaluation of metacognitive knowledge” (represented as a red square in the diagrams of Table 3) whereas the learning objective of “intervention” was defined to the level of **“**creation of procedural knowledge” (represented as a green square in the diagrams of Table 3). The coloured circles in the diagrams show the actual level of knowledge expressed by the individual GP within the four learning objectives: overall (yellow), “identification” (grey), “referring” (red), and “intervention” (green).

### 3.3. Stage 3: A Description of Each Major Component of the Cases

In order to analyze the individual challenges and learning processes, three steps were taken.

1. Each case was structured using the individual GPs' own formulated learning objectives before the intervention began, halfway, and finally after the project ended after two years.
2. Each individual learning objective was then related to the overall learning objectives of the project and colour-coded according to the revised taxonomies framework.
3. The individual learning processes were each depicted in a table showing the revised taxonomy model.

The participants were allowed to describe as many learning objectives for their participation in the project as they wanted. They all defined 2, 3, or 4 learning objectives. At each level of the individual development, the learning objectives were categorized as within the applied taxonomy. If the participant said “I want to become better at spotting children in need,” this was perceived as working with the project's learning objective of identification and then categorized according to the taxonomy used. If the learning reached the level of strengthening the medical professional identity within the specific self-identified learning objectives they were working with, it was colour-coded yellow and plotted to the knowledge level they had reached within this specific area. If the GP reached the knowledge level described in the curriculum, this can be seen plotted as a circle within the square of the same colour. The analysis is summarized in Table 3.

### 3.4. Stage 4: Vignettes which Describe Particular Episodes

Quotes from the final interviews with the six participants are included in order to show how the participants expressed their perception of the individual processes of professional learning as taking part in a educational supervision group.

*Ann*: When you are sitting in a group and hear that you are not the only one who experiences problems with saying some things and get the consultation going regarding a difficult issue, and that the others dared, or they did not dare for that matter, then I say: So what? We all have the same problems: it's not THAT different among us.

*Brian*: The supervision gives thoughtfulness and reflection. I mean, you get time to reflect, that's what it aims at, and that is really rewarding. When you think of how we work, this is the way we learn. Because we work so spread out and at the same time together anyway.

*Claire: *I don't really think it has been good to bring up my own cases, there has been a lot of good things in the other participants; cases (…), but I think I often got advice instead of reflections, and I just can't take that.

*David*: To bring up stuff in the group has been connected with a certain element of: have I presented it well enough? The supervisor has been saying don't think about how you say it, just say it. But he can do that from here to Jerusalem, I would never do that, never.

*Erica*: Participating in the project has sharpened my attention; I have a completely new attention on children and families and remember to include the children even if the issue is something else. I have become more confident and dare to say let us see what happens. I now know that some things are not my business and that I as a GP cannot save the world; we do have limitations. You can support the families, bring attention to the problem, and then pass the problem on to a relevant authority and that may be the end of what I can do.

*Fred*: I now have an overview of our collaborators and what I can refer to and how to do it. I know what I can use the children ward for, if I get a suspicion I can admit them for observation, and I know what to write. I have developed a language.

### 3.5. Summary of Main Findings

The aim of the paper was to describe the individual processes of professional learning and challenges among GPs as they take part in educational peer-group supervision. Combining the distinction between developing professional behaviour and professionalism [7] with the applied taxonomy [16], we have defined the development of professional behaviour as moving towards *a gain of factual, conceptual, and procedural knowledge to the level of application.* Within this definition development of professionalism additionally implies a gain of meta-cognitive knowledge to the level of analysis, evaluation, and creativity.

 The analysis has shown that all GPs developed their professional behaviour and many of them strengthened their professional identity in this domain towards a changed professionalism. Most of the participants emphasized the positive experience of sharing their worries with the children and the families themselves, demonstrating that they care for the patients. If you look at the diagrams of learning (Table 3), you can see that some of the GPs' learning processes were very linear/convergent (e.g., Brian). Others had a more complex/divergent learning process, starting with a relatively simple objective but gradually realizing how multifaceted the issue was after the first year, leading to a development of understanding new perspectives or action possibilities at the end of the project (e.g., Erica).

The new and inexperienced GP (David) and the most experienced GP (Claire) did not gain what they had hoped. They both developed, but David seemed to have set his expectations too high or perhaps wished for more complicated learning to happen. Being a novice GP, David, expressed the need to develop his own experiences rather than what he described as “transferring knowledge” from the more experienced GPs. Claire progressed to more complex competences but still not to the degree she had hoped for. In relation to our definition of professionalism, her application of metacognitive knowledge reached the level of “analysis” but not “evaluation and creativity.”

Two of the GPs (Fred and Erica) spontaneously described their new development of new thinking or professional language.

## 4. Discussion

By participating in educational peer-group supervision for two years focusing on child cases, some GPs did not gain what they expected and only a few developed their professionalism to the extent to which the project had aimed.

We cannot be certain about the reasons for different developments among the GPs, but one explanation could be the GPs' different experiences, both in terms of clinical experiences and experiences in the use of supervision as a CPD method.

Another explanation could be that, although the participants received the same intervention, the two from the rural group (Erica and Fred) were supervised by a clinical psychologist/child expert instead of a GP, leading to potentially different perspectives in the individual supervision sessions. The focus of the study was not the quality of the supervision. But in the overall evaluation at the end of the study, all participants expressed that they had gained tremendously from the supervision in the development of their professional skills. They felt able to identify children in need, as they felt able to define the specific initiatives these children required but had also gained the understanding of learning within this field as dependent on continuous learning, expressing that they felt in need of more training in identifying and working with partners in care [12].

### 4.1. Strengths and Limitations

The project design gave the possibility of following the participants over time, supporting the internal validity [15], as it is based on longitudinal triangulated data; not only the participants' responses in the interview situations, but also data from observations in group interaction, at teaching days, and analysis of the electronic portfolio designed for the project (the evaluation data collected is described in detail in [12].) In this paper we focus on the process of professional learning and challenges at the individual level. In another paper we have analysed the collective and interactive dimensions in depth [12] making these perspectives more implicit than explicit in the analysis in this paper.

The six cases represent different gender, age, practice organization, geography, and previous experience with educational peer-group supervision. The participants, however, can all be described as white middle class, which is likely to have influenced the professional challenges presented. Our aim to describe individual learning processes called for the development of an analytical framework based on existing literature on taxonomy as well as medical professionalism.

The analytical model we have developed (Table 3) and used in this paper to describe the development of professional behaviour and professionalism did not account for the development beyond self-reported change. This model was only used to describe the professionalism and professional behaviour attained as set out for this specific project, focusing on children in need. The revised taxonomies framework we have used might not be appropriate for describing other aspects of a GP's professionalism [16, 17] or other clinical areas.

Other methods for data collection and analysis could have been used; for instance, we did not ask the participants to fill in learning style surveys and we could have chosen other parameters used to access adult learning. Our choices for data collection and analysis were affected by the overall aim of the project [15] to strengthen the professionalism of the GPs working with paediatric cases.

Finally, we cannot predict the GPs' learning processes after the intervention or how the processes would have looked, if the intervention had lasted longer.

## 5. Conclusion

It has not been possible to identify any articles regarding individual learning processes for GPs working with clinical challenges in educational supervision groups. We therefore suggest that this paper is a contribution to an emerging field, demonstrating the need to focus on individual learning trajectories in a group learning context.

The study took its point of departure in a project focusing on children in need. It is however our expectation that the mechanisms in the educational peer group, including the individual outcome, is transferable to educational peer supervision groups with other clinical foci.

The results of the study suggest that there might be several elements playing a significant role in the development of professional behaviour and professionalism for the individual GP participating in an educational peer supervision group: the composition of the supervision groups in terms of participants' clinical experiences and experiences with supervision as a CPD method, as well as the professional background of the supervisor. This we hope will be part of the considerations for any course organizer planning future educational supervision as part of CPD.

The paper also suggests an analytical framework to describe the individual GPs' development of professional behavior and professionalism when working in educational peer-group supervision.

It is our hope that the analytical model we have developed in this paper will encourage other researchers to further studies of the impact of educational peer group supervision at an individual level. 

##  Conflict of Interests

This study was supported by the Danish Ministry of Interior and Health.

## Figures and Tables

**Table 1:** Description of the cases.

Cases	Age	Geography	No. of years in practice	Practice organization	Previous experience with supervision as cpd	Supervisor professional background
Ann	35	Urban	2	Shared practice, work part time	No	GP
Brian	45	Urban	10	Solo practice	No	GP
Claudia	58	Suburban	30	Solo practice	Yes	GP
David	38	Suburban	0	Shared practice	No	GP
Erica	45	Countryside	8	Shared practice	No	Child psychologist
Fred	42	Countryside	4	Shared practice	No	Child psychologist

**Table 2:** Theoretical model (adapted from [18]).

Cognitive Processes						
The knowledge Dimensions	1: Remember	2: Understand	3: Apply	4: Analyze	5: Evaluate	6: Create
A: Factual						
B: Conceptual						
C: Procedural						
D: Metacognitive						

**Table 3:** Summary of analysis.